# Correction: Rapid access to diverse, trifluoromethyl-substituted alkenes using complementary strategies

**DOI:** 10.1039/c8sc90109k

**Published:** 2018-05-25

**Authors:** James P. Phelan, Rebecca J. Wiles, Simon B. Lang, Christopher B. Kelly, Gary A. Molander

**Affiliations:** a Roy and Diana Vagelos Laboratories , Department of Chemistry , University of Pennsylvania , 231 South 34th Street , Philadelphia , Pennsylvania 19104-6323 , USA . Email: gmolandr@sas.upenn.edu

## Abstract

Correction for ‘Rapid access to diverse, trifluoromethyl-substituted alkenes using complementary strategies’ by James P. Phelan *et al.*, *Chem. Sci.*, 2018, **9**, 3215–3220.



## 

The authors regret that in the original article the Electronic Supplementary Information footnote included additional incorrect CCDC numbers for the crystal data reported. The correct CCDC number for the crystal data reported in the article is 1811877. The downloadable crystal data for the manuscript were also updated with the correct file.

The Royal Society of Chemistry apologises for these errors and any consequent inconvenience to authors and readers.

